# P-884. A Scenario-Based Survey of Clinician Recommendations for Follow-up Blood Cultures in Patients with Varying Risk of Persistent Gram-Negative Bacteremia

**DOI:** 10.1093/ofid/ofae631.1075

**Published:** 2025-01-29

**Authors:** Roberta Monardo, Larry Park, Ilan Schwartz, Marco Ripa, Joshua T Thaden, Vance G Fowler, Sonali Advani, Stacey Maskarinec

**Affiliations:** Vita-Salute San Raffaele University, Milan, Lombardia, Italy; Duke University Department of Medicine, Durham, North Carolina; Duke University, Durham, NC; San Raffaele University Hospital, Milan, Lombardia, Italy; Duke University School of Medicine, Durham, NC; Duke University Medical Center, Durham, NC; Duke University School of Medicine, Durham, NC; Duke University Medical Center, Durham, NC

## Abstract

**Background:**

Studies examining the impact of follow-up blood cultures (FUBCs) in patients with gram-negative bacteremia (GNB) have shown mixed results. In practice, FUBCs are variably obtained. Understanding practice variation and motivation for ordering FUBCs is important to determine how providers stratify patients at high risk for persistent GNB.

**Figure 1:**
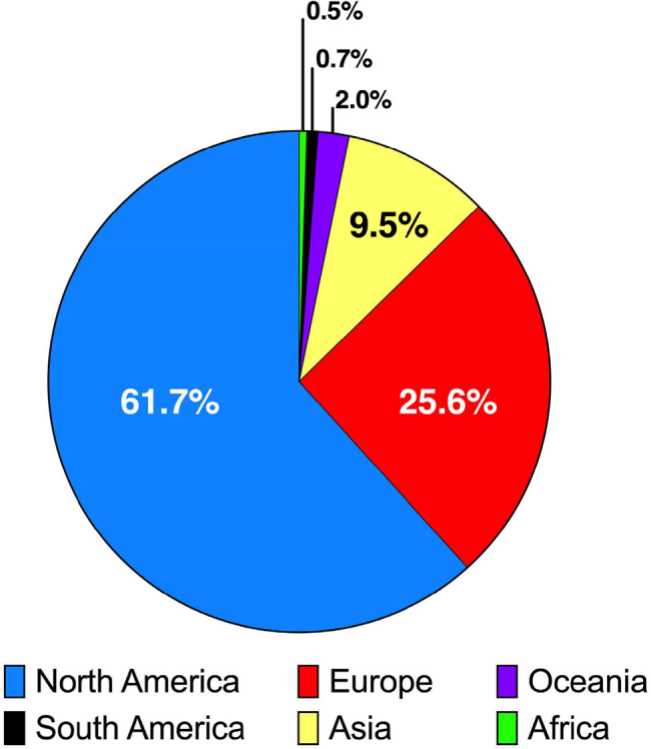
Global distribution of survey respondents (n=864).

**Methods:**

We surveyed clinicians (physicians, pharmacists, clinical microbiologists, physician assistants, nurses, medical trainees) globally between 2/21/24-4/9/2024 to understand when they would obtain FUBCs in patients with GNB using 10 hypothetical patient scenarios. Scenarios were generated by varying age, clinical status after 48 h of antibiotics, and risk of persistent GNB based on our published AIMS scoring tool (*A*ntibiotics, *I*nfection source, *M*edical conditions, *Serratia*). The survey contained 4 low-risk cases (AIMS 0-1), 3 intermediate-risk cases (AIMS 2-3), and 3 high-risk cases (AIMS 4-6). This anonymous web-based survey was distributed through listservs, email, and social media.

**Table 1:**
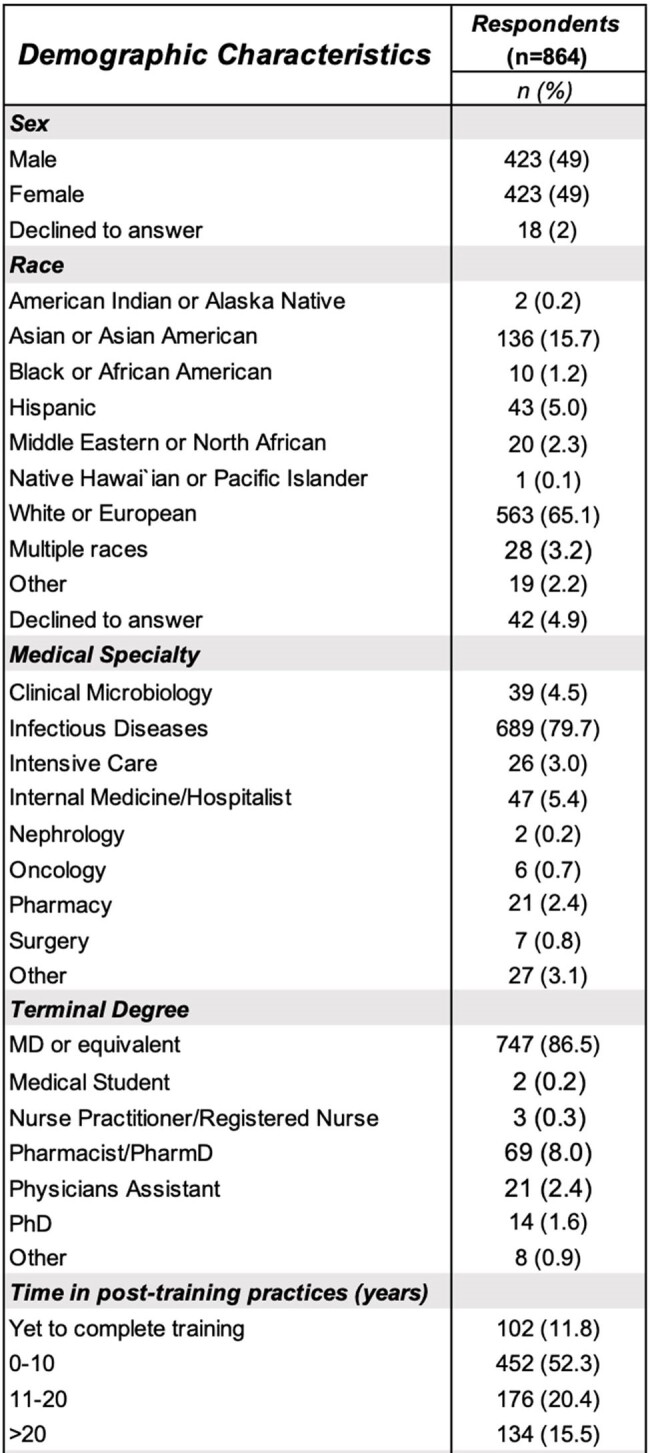
Demographic characteristics of survey respondents (n=864).

**Results:**

In total, 864 respondents from 50 different countries and 6 continents (Figure 1) opened the survey and provided 791 recommendations on FUBC ordering (791/864; 91.5%). Respondent demographics are described in Table 1. Most respondents were physicians (86.5%) specializing in infectious diseases (79.7%) who were within 10 years of training completion (52.3%); a plurality managed least 6-10 cases of GNB/month (22.7%). FUBCs were frequently recommended, with >40% of respondents recommending FUBCs in 7/10 scenarios. Fair inter-respondent agreement was seen across all scenarios (Fleiss kappa 0.27), with notable agreement for cases with high AIMS scores (Fleiss kappa 0.24) (Figure 2). Patient age and clinical status after antibiotic administration were not associated with FUBC recommendations.

**Figure:**
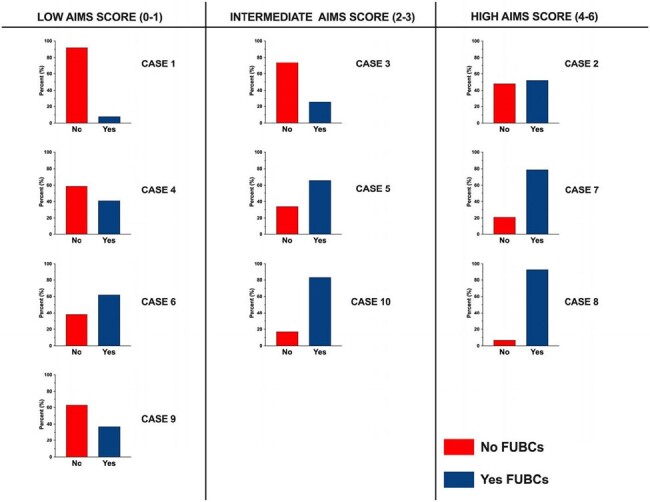

Survey results of FUBC recommendations by individual case scenario (1-10) stratified by low, intermediate, and high risk AIMS scores. Bar graphs represent percentage of recommendations in favor (yes) or not in favor (No) of FUBCs in patients with GNB.

**Conclusion:**

In this survey study, clinicians risk stratified hypothetical patients with GNB when assessing the need for FUBC. There was fair agreement among clinicians that high risk patients should get FUBCs. Future studies should focus on variables which prompt FUBC ordering in low-risk patients, an area of disagreement among responders, and ideally in the form of randomized clinical trial.

**Disclosures:**

**Joshua T. Thaden, MD, PhD**, National Institutes of Health K08 AI171183 (Thaden): Grant/Research Support **Vance G. Fowler, MD, MHS**, Affinergy: Advisor/Consultant|ArcBio: Stocks/Bonds (Private Company)|Armata: Advisor/Consultant|Astra Zeneca: Advisor/Consultant|Astra Zeneca: Grant/Research Support|Basilea: Advisor/Consultant|Basilea: Grant/Research Support|ContraFect: Advisor/Consultant|ContraFect: Grant/Research Support|Debiopharm: Advisor/Consultant|Destiny: Advisor/Consultant|EDE: Grant/Research Support|Genentech: Advisor/Consultant|Genentech: Grant/Research Support|GSK: Advisor/Consultant|Janssen: Advisor/Consultant|Karius: Grant/Research Support|MedImmune: Grant/Research Support|Merck: Grant/Research Support|sepsis diagnostics: Patent pending|UptoDate: Royalties|Valanbuio: Stocks/Bonds (Private Company)|Valanbuio: Stocks/Bonds (Private Company) **Sonali Advani, MBBS, MPH, FIDSA**, Biomerieux: Advisor/Consultant|GSK: Advisor/Consultant|Locus Biosciences: Advisor/Consultant|Sysmex America: Advisor/Consultant **Stacey Maskarinec, MD, PHD**, National Institutes of Health K23 HL159275 (Maskarinec): Grant/Research Support

